# Elastic Modulus of Muscle and Tendon with Shear Wave Ultrasound Elastography: Variations with Different Technical Settings

**DOI:** 10.1371/journal.pone.0044348

**Published:** 2012-08-31

**Authors:** Brian Chin Wing Kot, Zhi Jie Zhang, Arthur Wai Chun Lee, Vivian Yee Fong Leung, Siu Ngor Fu

**Affiliations:** 1 Department of Rehabilitation Sciences, The Hong Kong Polytechnic University, Hung Hom, Kowloon, Hong Kong SAR, China; 2 Department of Imaging and Interventional Radiology, Prince of Wales Hospital, The Chinese University of Hong Kong, Shatin, New Territories, Hong Kong SAR, China; Semmelweis University, Hungary

## Abstract

Standardization on Shear wave ultrasound elastography (SWUE) technical settings will not only ensure that the results are accurate, but also detect any differences over time that may be attributed to true physiological changes. The present study evaluated the variations of elastic modulus of muscle and tendon using SWUE when different technical aspects were altered. The results of this study indicated that variations of elastic modulus of muscle and tendon were found when different transducer’s pressure and region of interest (ROI)’s size were applied. No significant differences in elastic modulus of the rectus femoris muscle and patellar tendon were found with different acquisition times of the SWUE sonogram. The SWUE on the muscle and tendon should be performed with the lightest transducer’s pressure, a shorter acquisition time for the SWUE sonogram, while measuring the mean elastic modulus regardless the ROI’s size.

## Introduction

The biomechanical properties of the musculoskeletal system are difficult to assess because these structures consist of complex active or passive tissues [1]. When an electrical excitation occurs in the muscle fibers, a mechanical response will result, in the form of shortening, in addition to a modification of the mechanical properties, in the form of hardening. Understanding muscle mechanical properties is essential in clinical diagnosis and research on musculoskeletal injuries and movement-related disorders, and the application of this knowledge to patient care is central to rehabilitation [2].

Stiffness of soft tissues is clinically assessed with functional examinations (e.g., the manual muscle test), validated clinical scales (e.g., modified Ashworth scale), force measurements using handheld and isokinetic dynamometers, and surface and fine wire electromyography (EMG). While these measurements provide information that clinicians can use to track the changes in muscle function in their patients, some are subjective or unreliable [3], [4].

Recently, imaging techniques have been applied to investigate muscle function. Extensive research in the field of muscle activity measurements by MRI underscores the important role of muscle MR in neurophysiologic research, diagnosis, and therapy [5]–[7]. Magnetic resonance elastography (MRE) is a non-invasive elasticity imaging technique based on a phase-sensitive MR sequence that detects the propagation of shear waves generated by an external vibrator. Its applications cover several fields, including orthopedics, sports medicine, physical medicine and rehabilitation, endocrinology, and rheumatology; these mainly help to investigate the effects of treatment for patients with muscle spasticity by using quantitative analysis. However, the spatial resolution of this technique remains limited and the large size of mechanical vibrators constrains measurement conditions, which limits the application of MRE to the musculoskeletal system.

Shear wave ultrasound elastography (SWUE) is a new real-time diagnostic imaging technique with freehand capabilities that uses ultrasound to quantitatively assess tissue differences in stiffness. SWUE operates on transient elastography principles, by measuring the propagation of shear waves induced by vibrations applied at various frequencies, which are then used to estimate the elastic modulus of the relaxed and contracted quadriceps muscle group [8]. Subsequently, transient elastography was applied to muscles to quantify the anisotropic properties of muscle tissue [1].

Although SWUE has an acceptable reliability on the assessment of muscle and tendon [9], information regarding the technical aspect on the SWUE is still scarce, with a lack of consensus. The tissue compression might still be operator dependent, and even a slight touch of the skin might influence the elastography result [10]. Few studies on SWUE have reported the choice of the region of interest (ROI)’s size [1], [8], while its effect on SWUE measurements has not yet been investigated. SWUE enabled the computation of a quantitative elasticity map of an organ in just a few milliseconds [11], which differed from the 8–12 seconds acquisition time of the SWUE sonogram, suggested by manufacturer’s preferred setting.

Standardization on SWUE technical settings will not only ensure that the results are accurate, but also detect any differences over time that may be attributed to true physiological changes. The present study aimed to evaluate the variations of elastic modulus of muscle and tendon using shear wave ultrasound imaging when different technical aspects were altered: transducer’s pressure, ROI’s size, and acquisition time.

## Methods

### Ethics Statement

Informed, written consent was obtained from each subject. This study was approved by the Human Subject Ethics Subcommittee of the Department of Rehabilitation Sciences, the Hong Kong Polytechnic University.

### Subjects

Twenty healthy subjects (14 males, 6 females) were recruited in this study. Thigh muscles of 40 legs from 20 subjects were assessed by SWUE. Exclusion criteria for the healthy subjects included a clinical history of any knee pain/injuries. The age range of the subjects was 21–33 y (mean, 26.4±3.5).

### Equipment

All ultrasound examinations were performed with the Aixplorer**®** ultrasound unit in conjunction with a 4 to 15 MHz, 40-mm linear transducer (Supersonic Imaging, Aix-en-Provence, France).

### Muscle and Tendon Ultrasound Examination

All ultrasound examinations were performed by the same operator (BK) and the operator was blinded to elastic modulus measurements obtained during the scanning. In the ultrasound examination, subjects laid supine on the examination couch in a room set at 25°C, with the knee at 30° of flexion [12]. A custom-made ankle stabilizer was used to maintain the hip in neutral alignment. The subject laid in this position for five minutes before the commencement of the examination, to ensure the elastic modulus of muscle and tendon were measured at resting phase. The left and right rectus femoris (RF) and patella tendon (PT) were assessed separately. The scanning site for RF ultrasound was determined with reference to the recommended placement of surface electrodes for electromyography (EMG) [13].

Both the RF and PT were initially identified by conventional grayscale ultrasound. Transverse view of the RF was obtained by placing the transducer on the marked point. Once the muscle fibers were identified, the transducer was oriented 90° to obtain the longitudinal view. Longitudinal scan plane of the PT was performed by placing the transducer at the level of the distal patella, with the knee at 30° of flexion to straighten the tendon, avoiding anisotropy. SWUE mode was then activated to measure the elastic modulus of muscle and tendon. The transducer was stationed very lightly on top of a generous amount of coupling gel, perpendicularly on the surface of the skin. The transducer was kept motionless for 8 to 12 seconds during the acquisition of the SWUE sonogram.

A circle that delineated the region of interest (ROI) for the measurement of elastic modulus was placed at the center of the SWUE acquisition box on both the RF and PT. The diameter of the ROI was defined by the thickness of the RF and PT [14]. Maximum and mean values of Elastic modulus (in kPa) on the RF and PT within the ROI were estimated by the built-in specific quantification program.

### Transducer’s Pressure

**Figure 1:**
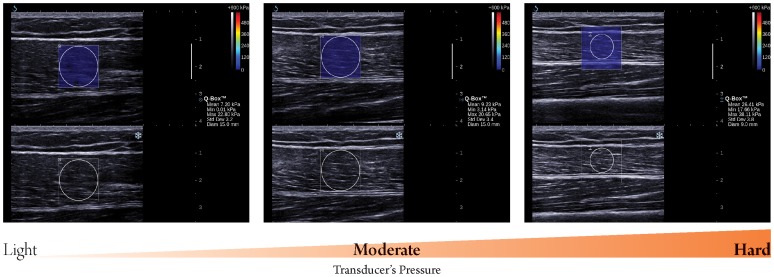
Longitudinal sonograms of rectus femoris (RF) taken with different transducer’s pressure. Upper images show color-coded box presentations of RF elasticity (stiffer areas were coded in red and softer areas in blue) superimposed on a longitudinal grey scale sonogram of RF, with the circle representing the region of interest and its corresponding elastic modulus demonstrating under Q-Box™ on the right. Bottom images show longitudinal grey scale sonograms of RF on the identical scan planes. Transducer’ pressure changed gradually from light to hard. The transducer was kept motionless for 8 to 12 seconds during the acquisition of the SWUE sonogram and ROI of 15 mm diameter was used.

**Figure 2:**
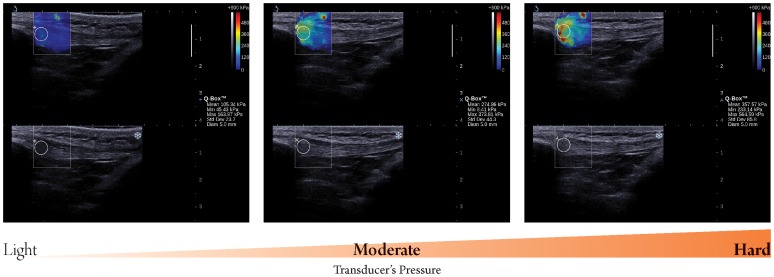
Longitudinal sonograms of patella tendon (PT) taken with different transducer’s pressure. Upper images show color-coded box presentations of PT elasticity (stiffer areas were coded in red and softer areas in blue) superimposed on longitudinal grey scale sonograms of PT, with the circle representing the region of interest and its corresponding elastic modulus demonstrating under Q-Box™ on the right. Bottom images show longitudinal grey scale sonograms of PT on the identical scan planes. Transducer’ pressure changed gradually from light to hard. The transducer was kept motionless for 8 to 12 seconds during the acquisition of the SWUE sonogram and ROI of 4 mm diameter was used.

The transducer’s pressure was categorized into 3 levels: light, moderate and hard. Light pressure was defined as placing the transducer very lightly on top of a generous amount of coupling gel on the surface of the skin without further applying artefactual areas of stiffness to the RF and PT. Hard pressure was presented by placing the transducer with a great force that could deform the thickness of the RF and PT, where moderate pressure was presented by placing the transducer with a gentle force that could just barely deform the thickness of the RF (Figure 1) and PT (Figure 2). When evaluating the difference on the elastic modulus of the RF and PT with different transducer pressures applied, the other two technical parameters were fixed at the preferred/suggested settings according to the manufacturer.

### ROI’s Size

**Figure 3:**
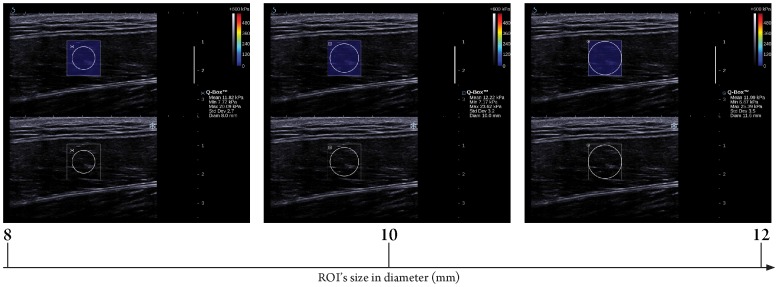
Longitudinal sonograms of rectus femoris (RF) with elastic modulus measured by various region of interest (ROI)’s sizes. Upper images show color-coded box presentations of RF elasticity (stiffer areas were coded in red and softer areas in blue) superimposed on a longitudinal grey scale sonogram of RF, with the circle representing the region of interest and its corresponding elastic modulus demonstrating under Q-Box™ on the right. Bottom images show longitudinal grey scale sonograms of RF on the identical scan planes. Transducer pressure applied was light for 8 to 12 seconds during the acquisition of the SWUE sonogram.

**Figure 4:**
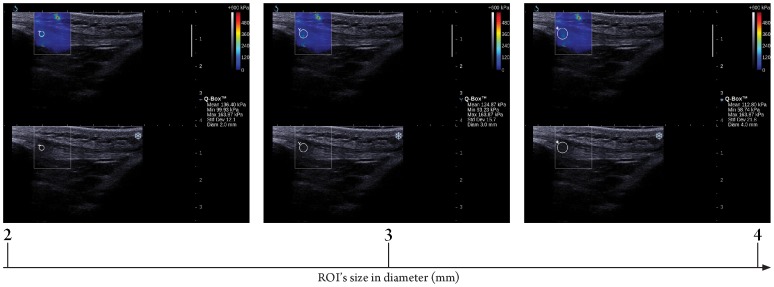
Longitudinal sonograms of patella tendon (PT) with elastic modulus measured by various region of interest (ROI)’s sizes. Upper images show color-coded box presentations of PT elasticity (stiffer areas were coded in red and softer areas in blue) superimposed on a longitudinal grey scale sonogram of PT, with the circle representing the region of interest and its corresponding elastic modulus demonstrating under Q-Box™ on the right. Bottom images show longitudinal grey scale sonograms of PT on the identical scan planes. Transducer pressure applied was light for 8 to 12 seconds during the acquisition of the SWUE sonogram.

ROI’s size was categorized into three levels (8, 10, and 12 mm diameter) for the RF (Figure 3) and three levels (2, 3, and 4 mm diameter) for the PT (Figure 4), respectively. Various sizes of ROI were placed in a concentric manner in the center of the SWUE acquisition box on both the RF and PT. When evaluating the difference on the elastic modulus of the RF and PT with ROI’s size used, the other two technical parameters were fixed with the preferred/suggested settings of the manufacturer.

### Acquisition Time of the SWUE Sonogram

Acquisition time of the SWUE sonogram was categorized into 4 levels: 5, 10, 15, and 20 seconds. The transducer was kept motionless for 5, 10, 15, and 20 seconds during the acquisition of the SWUE sonogram, and the corresponding elastic modulus of the RF and PT was estimated respectively. When evaluating the difference on the elastic modulus of the RF and PT with different acquisition times of the SWUE sonogram, the other two technical parameters were fixed with the preferred/suggested settings of the manufacturer.

### Statistical Analysis

The level of significance of the difference between the groups in the three investigated technical parameters (Transducer’s pressure, ROI’s size, Acquisition time of the SWUE sonogram) in the maximum and mean values of the elastic s modulus of the RF and PT were calculated by the Friedman Test with Dunn’s multiple comparison tests for post-hoc analysis. GraphPad InStat software was used for all statistical analyses (GraphPad Software Inc., San Diego, CA, USA).

## Results

A total of 40 RF and PT were evaluated with ultrasonography in the 20 healthy subjects. Results showed that there were significant differences (p<0.05, Table 1) in both the maximum and mean value of the elastic modulus of the RF and PT when different transducer’s pressure was applied. Further evaluation using post-hoc Dunn’s multiple comparisons tests indicated that light pressure gives a significantly smaller maximum and mean elastic modulus than when measuring with moderate and hard pressure, while moderate pressure gives a significantly smaller maximum and mean elastic modulus than when measuring with hard pressure.

**Table 1:** Comparison of the maximum and mean value of Elastic modulus of RF and PT when different transducer’s pressure was applied.

	Maximum value				Mean value			
	Mean±SD (kPa)			*p-value*	Mean±SD (kPa)			*p-value*
	Light	Moderate	Hard		Light	Moderate	Hard	
**Muscle**	40.12**±**23.45	46.29**±**25.00	56.64**±**28.67	<0.05	12.78**±**3.56	18.51**±**6.71	32.39**±**14.17	<0.05
**Tendon**	100.23**±**44.61	162.51**±**79.66	209.27**±**122.51	<0.05	69.80**±**32.14	107.33**±**53.63	134.57**±**73.75	<0.05

Results showed that there were significant differences (p<0.05, Table 2) in the maximum value of the elastic modulus of the RF and PT when different ROI’s size was used. Further evaluation of the muscle using post-hoc Dunn’s multiple comparisons tests indicated that a significantly smaller maximum elastic modulus of RF resulted from using the 8 mm diameter ROI’s size, than that of using the 10 mm and 12 mm diameter ROI’s size, while a significantly smaller maximum elastic modulus of RF was observed from using the 10 mm rather than the 12 mm diameter ROI’s size. Further evaluation of the tendon using post-hoc Dunn’s multiple comparisons tests indicated that a significantly smaller maximum elastic modulus of PT resulted from using the 2 mm diameter ROI’s size, than that of using the 3 mm and 4 mm diameter ROI’s size. However, there was no significant difference in the mean value of the elastic modulus of the RF and PT when different ROI’s size was used.

**Table 2:** Comparison of the maximum and mean value of Elastic modulus of RF and PT when different ROI’s size was used.

	Maximum value				Mean value			
	Mean ± SD (kPa)			*p-value*	Mean ± SD (kPa)			*p-value*
	8 mm	10 mm	12 mm		8 mm	10 mm	12 mm	
**Muscle**	25.92**±**9.22	30.22**±**14.21	36.68**±**18.86	<0.05	12.78**±**3.53	12.71**±**3.40	12.68**±**3.55	0.92
	**2 mm**	**3 mm**	**4 mm**		**2 mm**	**3 mm**	**4 mm**	
**Tendon**	86.67**±**43.58	92.20**±**42.35	97.18**±**43.53	<0.05	72.48**±**36.45	69.98**±**33.58	70.00**±**32.11	0.47

There were no significant differences in both the maximum and mean value of the elastic modulus of the RF and PT with different acquisition times of the SWUE sonogram were used (p>0.05).

## Discussion

SWUE is a new real time ultrasound imaging mode that quantitatively measures the elastic modulus of local tissue. This new mode appears to be a promising tool to improve understanding of the elastic properties of musculoskeletal tissues, which facilitates diagnosis and evaluation of degenerative myopathies, and assists in determining the best rehabilitation program for sports injuries patients, stroke patients and diabetes [8], [14]. SWUE demonstrates not only the traditional colour-coded images of tissue hardness, superimposed on a grayscale sonogram, on all striated muscles and superficial tendons (provided that the transducer can be placed on their surface), but also quantitatively presents the colour scale with the maximum and mean elastic modulus values expressed in kPa. Previous studies have demonstrated that SWUE is capable of estimating the elastic modulus of muscle, but to the best of our knowledge, there has been no standardization of SWUE’s technical settings, which has hindered its clinical applications. The results of this study indicated that variations of elastic modulus of muscle and tendon were found when different transducer’s pressure and region of interest (ROI)’s size were applied, which might possibly reflect the presence of measurement error due to altered/unstandardized technical settings of SWUE.

There were significant differences in both the maximum and mean value of the elastic modulus of the RF and PT when different transducer pressures were applied. In addition, the elastic modulus of the RF and PT increased with increasing transducer’s pressure. With greater pressure exerted on the skin, stiffness of the PF and RT was affected since the sum total elastic modulus consists of the pressure by the external load, the underlying subcutaneous fat, as well as those of the muscle and tendon themselves. In contrast to the traditional ultrasound elastography technique, which requires deformation/compression of targeted tissues to produce strain within the tissue resulting in different grades of elasticity displayed over the grayscale sonogram [15], [16], SWUE produces an elastography sonogram based on the combination of a radiation force induced in a tissue by an ultrasonic beam and an ultrafast imaging sequence capable of capturing the real-time propagation of the resulting shear waves [11]. Therefore, no external load was required in SWUE, as the operator’s compression pressure strongly and directly affects the investigated tissues properties, leading to an error in the resulting elastic modulus. We support the manufacturer’s suggested technical setting that the operator should only induce light pressure with the transducer, at the surface of the skin with a generous amount of coupling gel.

Results of the present study showed that there were significant differences in the maximum value of the elastic modulus of the RF and PT when different ROI’s size was used. In addition, the maximum elastic modulus of the RF and PT increased with increasing ROI’s size. The shape of the ROI in the ultrasound unit was a circular structure by default, with a range of diameters (2–12 mm) for the measurement of the elastic modulus of the targeted region/tissue, placed at the center of the SWUE acquisition box. The larger the ROI’s size, the higher the chance to include the muscle fascia and dense collagen fiber, which accounted for the maximum value of the elastic modulus within the SWUE acquisition box. Previous studies with the application of SWUE on liver, breast and thyroid focused solely on the differentiation of the elastic modulus between benign and malignant lesions [17]–[19], therefore the size of the ROI was determined by the area of the suspected malignancy. However, in the musculoskeletal system, clinicians/physiotherapists are interested in understanding the degree of musculoskeletal spasticity and treat the entire affected musculoskeletal tissue as a whole [8]. In contrast to the maximum value of elastic modulus, no significant difference in the mean value of the elasticity modulus of the RF and PT was found, when different ROI’s size was used, due to the averaging of the elasticmodulus in the different sizes of ROI. Therefore, it is preferable to report the mean value of the elastic modulus of the muscle and tendon when using different ROI’s size across different scanning sessions.

No significant difference was found in both the maximum and mean value of the elastic modulus of the RF and PT when comparing different acquisition times of SWUE sonograms. Previous studies using SWUE had a great discrepancy on the determination of the acquisition time of SWUE sonograms, ranging from 5 seconds [14] to 10–20 seconds [19], which differed from the manufacturer’s suggested duration (8–12 seconds). Although there was no significant difference found in both the maximum and mean value of the elastic modulus of the RF and PT with increased acquisition time up to 20 seconds, the operator should be cautious of the transducer’s positioning since there may be a position shift of the transducer when acquisition time increases, which would affect the measurement of the elasticmodulus of the affected tissues since the imaged area may no longer be the original affected area.

The present study evaluated the variations of the elastic modulus of the muscle and tendon when different technical aspects were altered: transducer’s pressure, region of interest (ROI)’s size, and acquisition time, using shear wave ultrasound imaging. However, other factors such as the underlying complex mechanical properties of musculoskeletal tissues, and how neuromuscular diseases would affect the stiffness of musculoskeletal tissues, were not evaluated in the study. Further studies to establish the norm of the elasticmodulus of musculoskeletal tissues in healthy subjects, elite athletes and patients with various muscle injuries are suggested. Transducer’s pressures were subjectively applied in the present study, which limited the quantitative evaluation on its effect on pre-compression. Although all the ultrasound examinations were performed by a single operator, and constructive technique by focusing on the deformation of structures to ensure constant different pressures was applied, further studies on quantifying the transducer’s pressure applied and its effect on elastic modulus are suggested.

### Conclusions

SWUE is a useful imaging technique in evaluating the variations of the elastic modulus of the muscle and tendon. The SWUE on the muscle and tendon should be performed with the lightest transducer’s pressure, a shorter acquisition time for the SWUE sonogram, while measuring the mean Elastic modulus regardless the region of interest (ROI)’s size. Considerable action should be taken in the standardization of various technical settings before obtaining meaningful data for diagnosis and for guiding corrective therapy.
